# Prediction for blood lactate during exercise using an artificial intelligence—Enabled electrocardiogram: a feasibility study

**DOI:** 10.3389/fphys.2023.1253598

**Published:** 2023-10-25

**Authors:** Shu-Chun Huang, Chen-Hung Lee, Chih-Chin Hsu, Sing-Ya Chang, Yu-An Chen, Chien-Hung Chiu, Ching-Chung Hsiao, Hong-Ren Su

**Affiliations:** ^1^ Department of Physical Medicine and Rehabilitation, New Taipei Municipal Tucheng Hospital, Chang Gung Memorial Hospital, Taipei, Taiwan; ^2^ Department of Physical Medicine & Rehabilitation, Chang Gung Memorial Hospital, Linkou, Taiwan; ^3^ College of Medicine, Chang Gung University, Taoyuan, Taiwan; ^4^ Division of Cardiology, Department of Internal Medicine, Chang Gung Memorial Hospital, Linkou, Taiwan; ^5^ Department of Physical Medicine and Rehabilitation, Chang Gung Memorial Hospital, Keelung, Taiwan; ^6^ School of Traditional Chinese Medicine, College of Medicine, Chang Gung University, Taoyuan, Taiwan; ^7^ Taipei Private Tsai Hsing Senior High School, Taipei, Taiwan; ^8^ Department of Surgery, Thoracic and Cardiovascular Surgery Division, Chang Gung Memorial Hospital, Taoyuan, Taiwan; ^9^ Department of Nephrology, New Taipei Municipal TuCheng Hospital, Taipei, Taiwan; ^10^ Super Genius Aitak Co., LTD., Taipei, Taiwan

**Keywords:** exercise, convolutional neural network, long short-term memory, recurrent neural network, residual network

## Abstract

**Introduction:** The acquisition of blood lactate concentration (BLC) during exercise is beneficial for endurance training, yet a convenient method to measure it remains unavailable. BLC and electrocardiogram (ECG) both exhibit variations with changes in exercise intensity and duration. In this study, we hypothesized that BLC during exercise can be predicted using ECG data.

**Methods:** Thirty-one healthy participants underwent four cardiopulmonary exercise tests, including one incremental test and three constant work rate (CWR) tests at low, moderate, and high intensity. Venous blood samples were obtained immediately after each CWR test to measure BLC. A mathematical model was constructed using 31 trios of CWR tests, which utilized a residual network combined with long short-term memory to analyze every beat of lead II ECG waveform as 2D images. An artificial neural network was used to analyze variables such as the RR interval, age, sex, and body mass index.

**Results:** The standard deviation of the fitting error was 0.12 mmol/L for low and moderate intensities, and 0.19 mmol/L for high intensity. Weighting analysis demonstrated that ECG data, including every beat of ECG waveform and RR interval, contribute predominantly.

**Conclusion:** By employing 2D convolution and artificial neural network-based methods, BLC during exercise can be accurately estimated non-invasively using ECG data, which has potential applications in exercise training.

## 1 Introduction

Lactate is produced as a byproduct of glycolysis during exercise. The production of lactate in working muscles is accelerated, especially during high-intensity exercise. Serial measurements of blood lactate concentration (BLC) during an incremental exercise test (INC) can determine two important thresholds: the lactate threshold and the onset of blood lactate accumulation. These thresholds are closely associated with metabolic and respiratory parameters (Esteve-Lanao et al., 2005; Seiler and Kjerland, 2006). These two thresholds are significant considerations in prescribing aerobic exercise, as training intensity between them effectively enhances fitness (Londeree, 1997). Additionally, BLC aids in understanding the metabolic adaptation to exercise. It decreases at the same absolute intensity after endurance exercise training (Donovan and Brooks, 1983). Despite its usefulness, the measurement of BLC currently necessitates blood sampling, and a convenient non-invasive alternative method is not yet available.

Electrocardiogram (ECG) changes as exercise intensity and duration vary. The normal ECG response to exercise includes the following: decease in RR interval, ST segment and J point depression, shortening of QT interval, minimal shortening of QRS duration (Kligfield and Lauer, 2006), slight decrease in R-wave amplitude (Myers et al., 1985), and increase in T-wave amplitude (American College of Sports Medicine, 2018). Since blood lactate concentration (BLC) also changes during exercise, it might be possible to estimate BLC by analyzing the ECG.

An electrocardiogram (ECG) is a graphical representation of the heart’s electrical activity, displaying voltage changes over time (Theresa, 2009). It is obtained by placing electrodes on the skin. Traditional ECG analysis primarily involves signal-noise filtering, segmentation, and manual feature extraction (Somani et al., 2021). In recent years, machine learning techniques, including support vector machines and linear discrete analysis, have been widely utilized in ECG analysis. However, these methods had certain limitations until the advent of deep learning (Miotto et al., 2018). Deep learning has significantly advanced the field of artificial intelligence (AI) and has found extensive applications in ECG analysis (LeCun et al., 2015; Mincholé et al., 2019; Rim et al., 2020; Nasir et al., 2021; Barneih et al., 2022; Qatmh et al., 2022). Given that an ECG is a time-based signal, one-dimensional convolutional neural network (1D CNN) (Krizhevsky et al., 2017) or recurrent neural network (RNN) (Sherstinsky, 2018) methods are commonly employed for analysis. In recent times, the combination of 2D CNN-based methods with RNN-based approaches has emerged, demonstrating superior results, particularly in automatic arrhythmia diagnosis (Zheng et al., 2020). The current study introduces modifications to existing deep learning methods and applies them for the first time to predict blood lactate concentration (BLC) through exercise ECG analysis.

**FIGURE 1:**
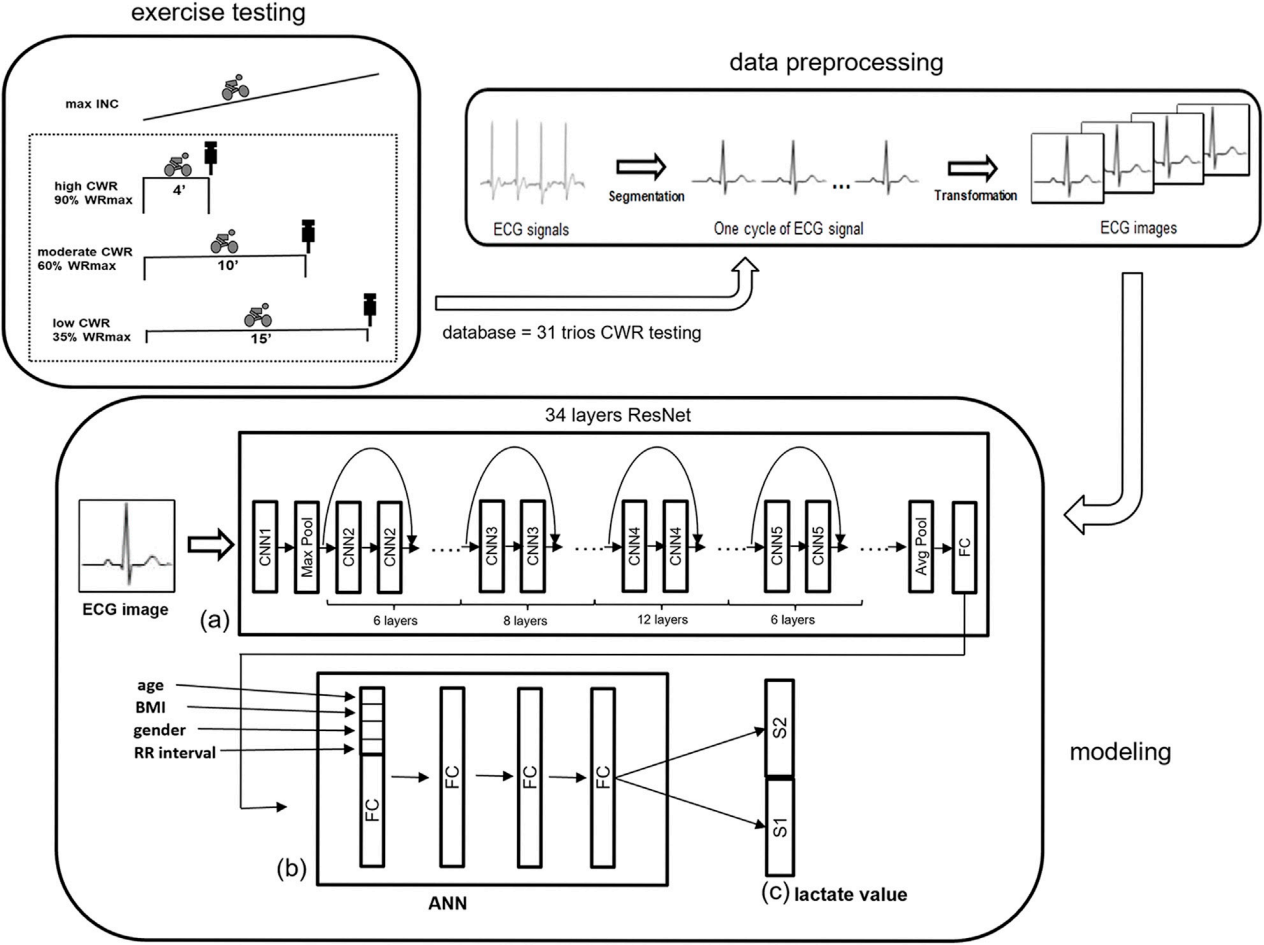
An illustration of the overall analytical flow path of ECG data. **(A)** A 34-layer ResNet is used to obtain the ECG waveform 2D feature, and **(B)** ANN is used to combine the one-beat ECG waveform with anthropometric information (age, BMI, and sex) and RR interval. **(C)** S1 and S2, of the last layer are binary classifications for BLC. Both S1 and S2 use binary to represent integers and floating-point numbers respectively. ANN = artificial neural network; INC = incremental exercise testing; ResNet = residual network.

Our hypothesis is that BLC can be predicted using ECG data, including waveform and RR intervals. In this study, we aimed to estimate BLC by analyzing exercise ECG data using a novel hybrid mathematical model based on deep learning. This model combines convolutional neural network (CNN) and artificial neural network (ANN), resulting in two components. The first component is a CNN spatial model used to address image-related challenges in analyzing 2D beat-to-beat ECGs. The second component involves an ANN model utilized for analyzing RR intervals and anthropometric data (Figure 1).

## 2 Methods

Thirty-one healthy male and female participants between 20 and 50 years of age were recruited using convenience sampling. Those with cardiovascular disease were excluded. The experimental protocol was approved by the Institutional Review Board of Chang Gung Memorial Hospital (102–2960B). All subjects provided written informed consent after receiving oral and printed explanations of the experimental procedures. This study was conducted in accordance with the ethical standards of the Declaration of Helsinki. The participants were recruited between 1 September 2013, and 31 May 2015. The authors had access to information that could identify individual participants during or after data collection.

### 2.1 Cardiopulmonary exercise test and blood lactate measurement

Each participant visited the exercise laboratory for four times within 1 week to receive cardiopulmonary exercise tests on a cycle ergometer (Ergoselect 150P, Ergoline, Germany): one incremental exercise test (INC) and three constant work rate (CWR) tests. The CWR tests were of low, moderate, and high intensities. Each subject was instructed to refrain from exercising for 12 h before each test. The INC comprised 1 min of unloaded pedaling followed by an incremental increase in the work rate of 15 W per minute until exhaustion; thereby, the maximal work rate was determined. The maximal V’O_2_ was defined using the following criteria: i) VO_2_ increased by < 2 mL/kg/min over at least 2 min, ii) HR exceeded 85% of its predicted maximum, iii) the respiratory exchange ratio exceeded 1.15, or iv) some other symptom/sign limitations (Balady et al., 2010). Subsequently, each subject performed three CWR exercise tests: 15-min low CWR at 35% maximal work rate (L), 10-min moderate CWR at 60% maximal work rate (M), and 4-min high CWR at 90% maximal work rate (H). The low (35%), moderate (60%), and high (90%) CWR intensities were chosen based on three zones: below ventilatory threshold 1 (VT1), between VT1 and VT2, and above VT2 (Esteve-Lanao et al., 2005; Seiler and Kjerland, 2006). In the majority of healthy people, VT1 occurs at 40%–60% of maximal V’O_2_ (Mezzani et al., 2009). VT2 has been reported to be 61.3%–85.4% of maximal V’O_2_ in healthy participants (Dekerle et al., 2003). Minute ventilation (V’E), oxygen uptake (V’O_2_), and carbon dioxide production (V’CO_2_) were measured breath by breath using a computer-based system (MasterScreen CPX, Cardinal Health, Germany). Heart rate (HR) was determined from the RR interval of a 12-lead ECG (CardioSoft, GE, Milwaukee, WI, United States). Arterial blood pressure was measured every 2 min using an automatic blood pressure system (Tango, SunTech Medical, United Kingdom), and arterial O_2_ saturation was monitored continuously using finger pulse oximetry (model 9500, Nonin Onyx, Plymouth, MN, United States). End-exercise values were determined as the average of the final 15 s of exercise for both INC and CWR. In the CWR tests, venous blood was sampled mostly from an antecubital vein or, in a few cases, from the dorsal interosseous metacarpal vein for the BLC assay 30–60 s after the end of CWR exercise tests. Samples were collected in NaF/K3EDTA tubes and placed on ice. Whole blood was centrifuged within 90 min to obtain plasma, which was then stored at 4°C. The BLC was measured using the enzymatic method within 14 days after sampling (DXC880i).

### 2.2 Mathematical model for lactate estimation

A novel model was proposed to estimate BLC using ECG signals at the end of CWR testing. The model is based on the architecture of a CNN combined with an ANN to determine the best predictors for BLC (Somani et al., 2021). The proposed method can be divided into three parts: data preprocessing, modeling, and inference (Figure 1).

### 2.3 Data preprocessing

The ECG signal is one-dimensional time series data composed of continuous cardiac beats causing repetitive wave groups. The ECG signals were segmented into multiple one-beat signals according to their R peak and RR interval (Figure 1). Every single beat of a 1D lead II ECG signal, sampled at a rate of 200 Hz, was then converted into 2D ECG images as the analyzed data, which ensured the integrity of the original ECG data to the greatest extent (Zheng et al., 2020).

### 2.4 Modeling

We hypothesized that BLC is a function of ECG data, composed of a waveform pattern structure. The model can be considered as follows.

$$Y=f\left(X\right)$$

Y represents the BLC, X represents ECG, and f is the BLC model. Although approaching the problem as a linear model to estimate f may seem like the simplest option, the relationship between a vast amount of ECG data and BLC cannot be adequately estimated using a linear model alone. To develop a robust and efficient BLC model, multiple one-beat signals of the ECG data X were converted into 2D images using the residual network (ResNet) architecture to capture the waveform pattern. The ResNet extracted the waveform pattern feature, which was then input into an artificial neural network (ANN) with fully connected layers (Hardesty, 2017) architecture to train the waveform feature to correspond to BLC Y.

ResNet (short for “Residual Network”) is a deep neural network architecture introduced by Kaiming He, Xiangyu Zhang, Shaoqing Ren, and Jian Sun of Microsoft Research in 2015 (He et al., 2016a). It is a type of convolutional neural network (CNN) designed to overcome the problem of vanishing gradients that can occur in very deep neural networks. ResNet’s key innovation is the use of residual connections, which enable the network to learn residual functions rather than approximate direct mappings. This is achieved by introducing shortcut connections that bypass one or more layers of the network, allowing the gradient to flow more directly and reducing the likelihood of vanishing gradients. ResNet has achieved state-of-the-art results in various computer vision tasks, such as image classification, object detection (Goodfellow et al., 2016), and segmentation. Numerous studies have demonstrated its ability to train deep networks with high accuracy, making it a popular choice for many practical applications in industry and academia. Given the importance of physiological features represented by the variation in ECG data, we leverage ResNet to learn the relationship between BLC and ECG and extract critical features.

In our proposed method, 34-layer ResNet has been used to estimate the BLC model f instead of the linear model. The 34-layer ResNet has several advantages over shallower architectures (Goodfellow et al., 2016):

1. Improved accuracy: The 34-layer ResNet can achieve higher accuracy than shallower networks on many computer vision tasks. This is because the network is able to learn more complex features and better capture the underlying patterns in the data.
2. Reduced vanishing gradients: The use of residual connections in the 34-layer ResNet helps to alleviate the problem of vanishing gradients, which can occur in very deep neural networks. This allows the gradient to flow more directly and reduces the likelihood of the gradients becoming too small to update the weights effectively.
3. Faster training: The 34-layer ResNet can be trained faster than other very deep architectures due to the use of residual connections. This is because the residual connections enable the network to learn the residual function, which can be easier to optimize than direct mapping.

Overall, the 34-layer ResNet is a powerful architecture that has demonstrated state-of-the-art performance on many computer vision tasks. Its ability to learn complex features, reduce vanishing gradients, and train faster makes it a popular choice for many practical applications (Goodfellow et al., 2016).

Considering that the relationship between BLC and ECG signals may vary from person to person during exercise, anthropometric factors were added including age, body mass index (BMI), and sex for AI to learn to build the best BLC estimation model. The proposed ResNet-based model is illustrated in Figure 1. ResNet obtains the waveform features, and ANN combines the one-beat ECG waveform pattern feature from ResNet with anthropometric factors. Because each beat of the ECG waveform analysis loses the message of the RR interval, the RR interval is added to the ANN step.

The 34 layers-ResNet was used to acquire a 300-dimension feature vector from the last fully connected layer to model the spatial information of the ECG waveform data. After ResNet, the other four parameters, including age, BMI, sex, and RR interval, were added to the head of the feature vector. An ANN with a fully connected layer was added to incorporate the other four parameters with the feature vector from the ECG data after ResNet. Finally, two parts, S1 and S2, of the last layer are binary classifications for BLC. Both S1 and S2 use binary to represent integers and floating-point numbers respectively.

The binary number system uses only two digits, typically represented by 0 and 1. For example, the binary number 1011 represents the decimal number 11. To convert this binary number to decimal, we added up the values of the bits with a value of 1: 1 × 2^0 + 1 × 2^1 + 0 × 2^2 + 1 × 2^3 = 1 + 2 + 0 + 8 = 11.

In CNNs used for recognition and classification, the last layer typically represents the category to which input belongs using a binary value of 0 or 1. We leveraged this characteristic by converting the BLC into a binary value that could be represented in the same way.

For example, suppose the BLC is 5.2. We represented the integer part 5 as 00000101 in S1 and the decimal part 0.2 as 00000010 in S2. This allowed us to treat each BLC as a classification problem and train our algorithmic model using the features of CNNs.

By adopting this approach, we were able to use the benefits of CNNs to train our model as a classification problem, even though the task at hand involved estimating BLCs rather than categorizing images.

In addition, according to our previous research (Huang et al., 2019), high intensity (H) and low and moderate intensities (LM) have a distinct relationship between BLC (dependent variable) and independent variables. Thus, the present study continued to establish a mathematical model separately for the H and LM data.

### 2.5 Training and inference

The proposed model, including ResNet-34 and ANN, was implemented using the PyTorch framework and Python. Fourfold cross-validation (Kohavi, 1995) was applied to avoid overfitting and verify accuracy. The original ECG data was randomly partitioned into four equal-sized sub-datasets. Of the four sub-datasets, a single sub-dataset was preserved as the validation data to test the model, and the rest of the three sub-datasets were used as training data. The cross-validation process was repeated four times, with each of the four sub-datasets used once as the validation data. The four results were then averaged to produce a single estimation.

### 2.6 Weighting

**FIGURE 2:**
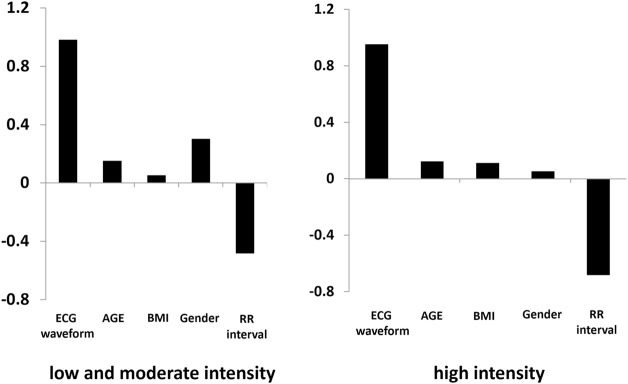
Absolute positive or negative weighting of each variable in determining the BLC.

**FIGURE 3:**
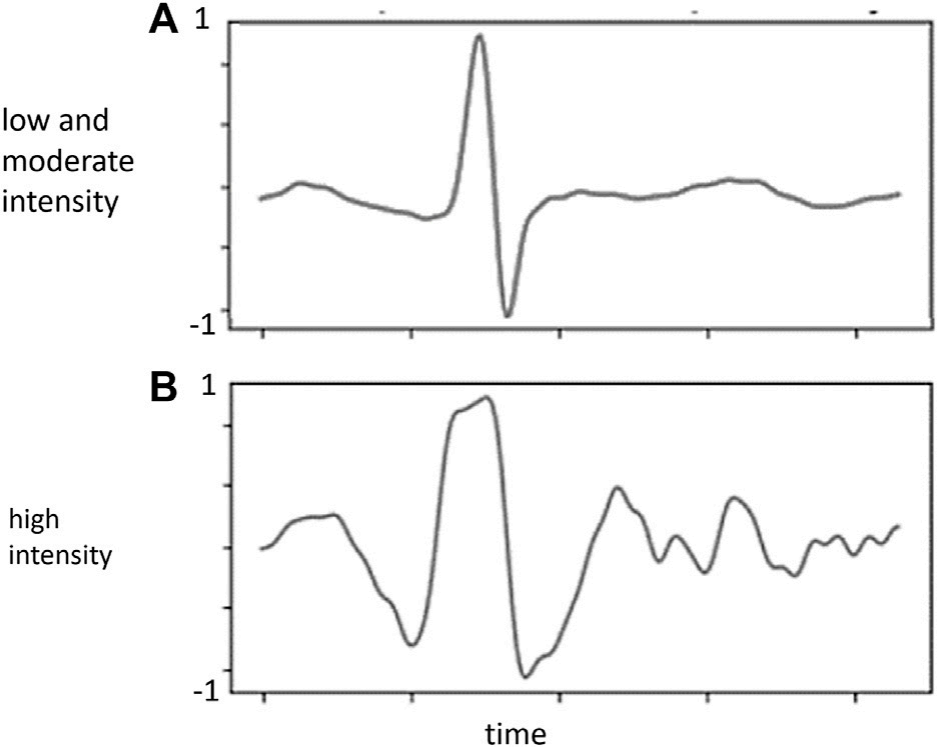
Normalized weighting (*Y*-axis) in each ECG waveform from the proposed model. **(A)** low and moderate intensity; **(B)** high intensity.

Weighting of the second layer in the (b) ANN model (Figure 1), which consists of 304 weights can be considered as the physiological parameters including RR interval, age, sex, BMI and ECG pattern trained from ResNet model when the proposed model is well trained. The first to fourth absolute positive or negative weights in Figure 2 represent age, sex, BMI, and RR interval. The other 300 weights are the weights of the ECG waveform, as shown in Figure 3. They were normalized from −1 to 1 to illustrate the relative weighting in a single ECG waveform. The total weighting of ECG is calculated by the average of 300 weights and is shown in the first column of Figure 2 as a comparison with age, sex, BMI, and RR interval. The formula for normalization is as follows:

Nonnegative ordinary weights:

$$\left\{w_{1},w_{2},w_{3},\ldots .,w_{t}\right\}$$ (1)

New weights:

$$w_{i}^{\prime }=y_{i}-mean\left(y_{i}\right)\;where\;y_{i}=\frac{w_{i}}{\sum_{i=1}^{t}w_{i}}$$ (2)

where w stands for the weighting of the second layer in the ANN model and $w_{i}^{\prime }$ stands for normalized weighting.

### 2.7 Statistics

Linear correlation and Bland–Altman plots were employed to demonstrate the validity of the estimated BLC. Descriptive statistics was used for the statistical analysis. Data are presented as the mean ± standard deviation.

## 3 Results

Fourteen men and seventeen women were recruited for the study. The participants’ age was 33 ± 9 years. The participants’ body height was 165 ± 9 cm. The body weight was 62.7 ± 11.6 kg. The BMI was 22.8 ± 2.7 kg/m^2^. The mean work rates at low, moderate, and high CWR were 66 ± 29 W, 107 ± 46 W, and 146 ± 66 W, respectively. The average BLC for the three intensities were 3.7 ± 2.3, 6.9 ± 4.2, and 10.4 ± 4.1 mM/L, respectively (Table 1).

**TABLE 1:** Physiological variables during incremental and constant work rate exercise testing.

INC		
maximal V’O_2_	(mL/min/kg)	30.1 ± 11.9
V’O_2_ at LT	(mL/min/kg)	21.9 ± 6.7
maximal WR	(watt)	187 ± 78
WR at LT	(watt)	119 ± 53
CWR		
WR at L	(watt)	66 ± 29
WR at M	(watt)	107 ± 46
WR at H	(watt)	146 ± 66
BLC at L	(mM/L)	3.7 ± 2.3
BLC at M	(mM/L)	6.9 ± 4.2
BLC at H	(mM/L)	10.4 ± 4.1

Mean ± standard deviation.

CWR, constant work rate; INC, incremental exercise test; LT, lactate threshold; WR, work rate.

L, low-intensity constant exercise test at 35% maximal work rate 15′; M, moderate-intensity constant exercise test at 60% maximal work rate 10′; H, high-intensity constant exercise test at 90% maximal work rate 4′.

### 3.1 Ideal number of consecutive QRS complexes to determine BLC

**FIGURE 4:**
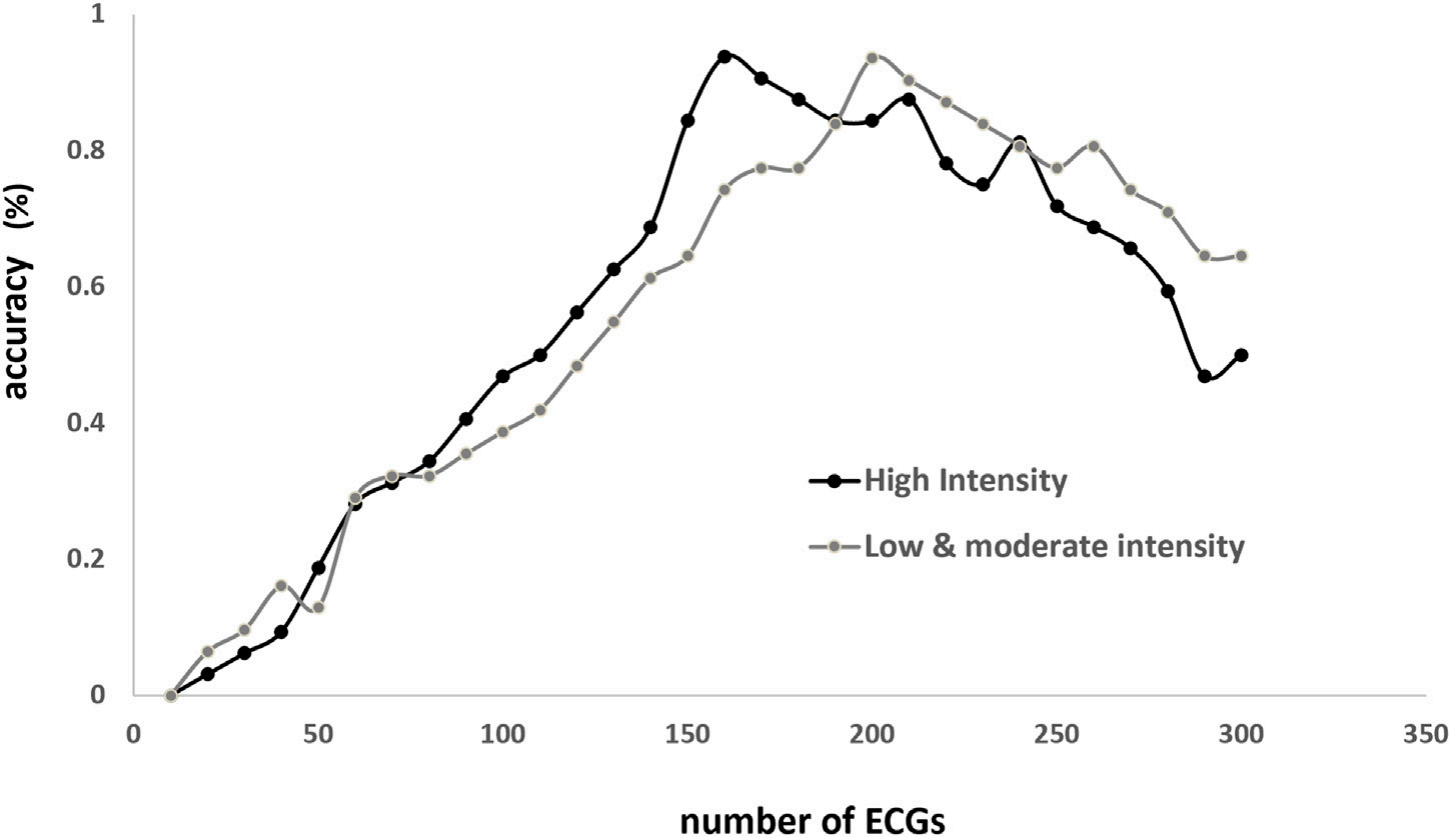
Relationship between accuracy (*Y*-axis) and number of beats (*X*-axis) employed to estimate blood lactate concentration (BLC; *X*-axis). Accuracy is the ratio of correct BLC estimation (person-time) to the total person-times under the same numbers of training times. Correct estimation is defined as the discrepancy <3% between estimated and true values.

To determine the number of consecutive QRS complexes to optimally predict BLC, the relationship between accuracy and the number of ECGs was analyzed under conditions of high, low, and moderate intensities (Figure 4). The results showed that the accuracy peaks were approximately 170 and 200 in H and LM intensities, respectively. Accordingly, 180 beats were employed in the following algorithm.

### 3.2 Accuracy of estimation

**FIGURE 5:**
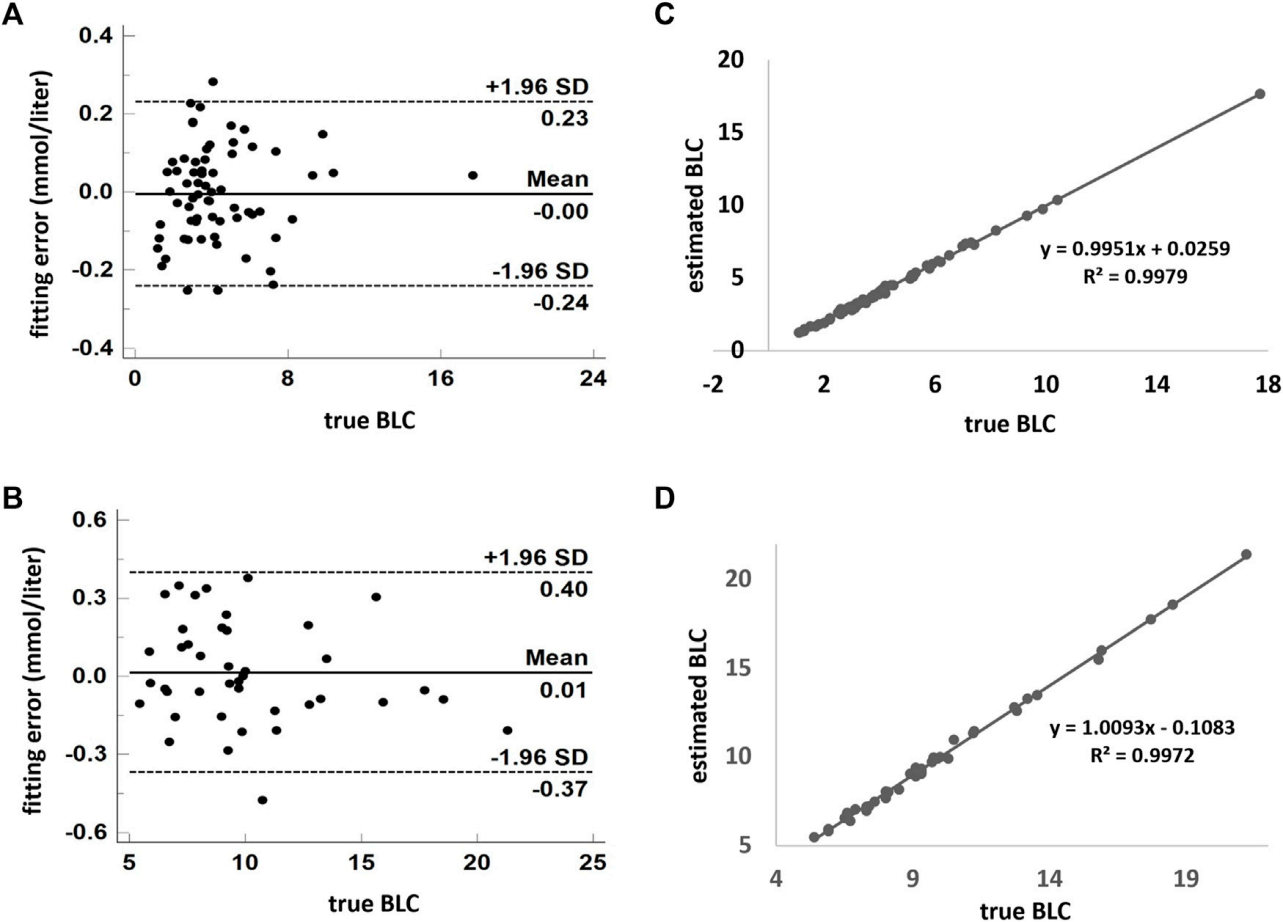
Model estimates of BLC compared with the measured value. **(A,B)** Combination of low- and moderate-intensity CWR tests. **(C,D)** High-intensity exercise. Scatterplots are demonstrated in (**B**,**D)**. Agreement using the Bland–Altman plot is shown in **(A,C)**. A and C show the fitting error between the estimated and measured values. The dark solid horizontal lines in each Bland–Altman plot represent average bias, whereas the dotted lines stand for average bias ±1.96 standard deviation (95% upper and lower limit). The standard deviation in A and C are 0.12 and 0.19 mmol/L, respectively. BLC: blood lactate concentration.

In both LM and H conditions, the models fit the data very well. The standard deviation of fitting error is 0.12 mmol/L in the former and 0.19 mmol/L in the latter (Figures 5A, B). The linear correlation coefficients are very close to one (Figures 5C, D). In addition, the fourfold cross-validation also showed that the data had high consistency, and the model was reliable and did not overfit (Table 2).

**TABLE 2:** Fourfold cross-validation.

Intensity	Fold				Mean	Variance
	1	2	3	4		
LM	99.58	99.65	99.34	99.68	99.56	0.02
H	98.98	98.78	99.23	99.32	99.01	0.06

### 3.3 Weighting analysis

Figure 2 shows the absolute weighting of each variable in determining the BLC. In both conditions, ECG was solely the major weighting factor. In the weighting analysis of the ECG waveform, BLC estimation is primarily contributed by signals around the R, S, and T waves (Figure 3).

## 4 Discussion

This is the very first study that utilizes exercise ECG to calculate the numerical values of BLC during exercise. In the past decades, resting ECG as big data has been used for very specific tasks in arrhythmias, valvulopathy, cardiomyopathy, ischemia, prognosticating health status, and event prediction (Somani et al., 2021). For example, a recent study showed that resting ECG may serve as a powerful tool for screening left ventricular dysfunction (Attia et al., 2019). The current study broadens the application of ECG. BLC could be obtained in a non-invasive and real-time manner by calculating data from a single-lead ECG through a deep learning-based approach.

CWR testing, a popular way to study exercise tolerance, has appeared for decades. CWR testing investigates a work rate domain likely to be encountered in everyday life (Wasserman et al., 2011). In the present investigation, responses to 31 trios of CWR exercise tests in 31 participants were utilized to establish a mathematical model to estimate the BLC from ECG data. The database consisted of low-, moderate-, and high-intensity exercise tests. The mathematical model is based on a combination of ResNet and ANN technology. Excellent fitting was achieved. The current study has several valuable findings for followers in the establishment of mathematical models for BLC estimation using deep learning. First, 170–200 consecutive beats are the optimal amount of data employed to estimate the BLC. Second, the 95% confidence interval of fitting errors are 0.24 and 0.38 in ML and H conditions, which are well acceptable for the purposes of specifying exercise training targets. Third, in weight distribution analysis showed that the ECG signal (including each ECG waveform and RR interval) was the major contributor for modeling. Other factors, including sex, age, and BMI, are extremely small. ECG data is sufficient to predict BLC. Fourth, the weighting analysis of ECG waveform shows that signals around R, S, and T waves play the main role.

### 4.1 BLC, a valuable index to manage aerobic exercise intensity

During aerobic endurance training, it is valuable to obtain the numerical value of BLC. Aerobic exercise intensity has been partitioned into three zones by VT1 and VT2, identified by gas exchange measurement during INC (Esteve-Lanao et al., 2005). The three intensity zones have distinctions in the duration to fatigue, sympathetic stress load, and motor unit involvement (Seiler and Kjerland, 2006). The production and elimination rates of blood lactate reached equilibrium in the moderate zone (between VT1 and VT2). VT2 is the highest possible intensity for obtaining a stable BLC, called the maximal lactate steady state (Dekerle et al., 2003; Seiler and Kjerland, 2006; Garcia-Tabar and Gorostiaga, 2018). The intensity of aerobic training in the moderate zone between VT1 and VT2 effectively improves fitness (Londeree, 1997). In the high zone above VT2, the BLC accumulates rapidly, and fatigue approaches. VT1 (also represented as anaerobic threshold or lactate threshold) and VT2 (also represented as respiratory compensatory point or onset of blood lactate accumulation) correspond to BLC approximately at 1–2 and 4 mM/L, respectively (Seiler and Kjerland, 2006).

A mathematical model was established in view of its application in the exercise industry. The algorithm of the current study could be applied to the threshold training model (Seiler and Kjerland, 2006) of cycling endurance training, in which the ideal intensity is to maintain the BLC in the range of VT1 and VT2, particularly for untrained people (Huang et al., 2019). The corresponding BLC values were 1–2 and 4 mM/L, respectively (Seiler and Kjerland, 2006). The SD of the fitting error in the LM is 0.12 mM/L, which is acceptable considering the width of the middle zone. One might argue that the current algorithm is limited by its division into LM and H intensity, potentially limiting its applicability. However, for aerobic endurance training, constant LM intensity training is typically preferred and performed. Therefore, a LM mathematical model should be sufficient. This is the main reason why we chose to separate the algorithm into two conditions. This strategy significantly improves accuracy without sacrificing practicality. In contrast, the H model is better suited for high intensity CWR exercise testing. The estimated value of BLC may be used as a criterion to help determine whether the testee approaches maximal exertion in case the gas analysis to measure V’O_2_ plateau is unavailable. A variety of BLC cutoff values have been proposed, and most of the criteria are approximately 8 mM/L (Van Remoortel et al., 2012).

The mathematical analysis of local muscle oxygen saturation signals, as measured by near-infrared spectroscopy, has been demonstrated to offer precise estimation of lactate-based exercise thresholds (Farzam et al., 2018; Batterson et al., 2023). A corresponding portable and wearable device is already commercially available. The methodology developed in the present study goes a step further by involving the computation of numerical values for BLC. This method can be applied to CWR exercise conditions. In addition to the cycle ergometer, the most prevalent scenario is treadmill exercise with a fixed incline and speed. Furthermore, some steppers are provided with CWR modes. Our model to estimate BLC would be relevant to a free ambulation CWR task in which ECG is measured. Prior to commercialization, the current algorithm, which relies on lead II signals, should undergo calibration using EKG signals obtained from a wearable device with a comparable sampling frequency. This typically involves the use of a pair of leads attached to the anterior chest.

### 4.2 Mixed models with ResNet and ANN

There are two primary types of neural networks in deep learning: CNN and ANN. These are the cornerstones of the deep learning revolution in popular applications. Here, we investigated the feasibility of combining these two models to integrate the advantages of the two underlying techniques.

Convolutional Neural Networks (CNNs) are primarily applied to computer vision and medical image analysis. When applied to a series of images or videos, CNNs enable AI systems to automatically extract features from these inputs to perform specific tasks, such as image classification or image segmentation (He et al., 2015; Badrinarayanan et al., 2017). There are numerous CNN frameworks available, and the ResNet architecture (He et al., 2016b; Somani et al., 2021) is employed in building the current feature model. In contrast, an Artificial Neural Network (ANN), which simulates the neural networks found in animal brains, serves as the most fundamental type of deep neural network. It consists of a series of fully connected layers suitable for statistical data analysis. In this study, four fully connected layers are utilized to integrate RR interval, age, sex, and BMI with the ECG patterns trained from the ResNet model. In the end, the ANN model is used to calculate the BLC for each individual based on their ECG data.

The present investigation adopted a novel method to analyze ECG data. It was built using the architecture of deep learning, which is currently the most important and mainstream method in AI. We assumed that the difference in the ECG waveform over time is an important feature that is highly correlated with the BLC. Therefore, the characteristics of the waveform over time were retained to the greatest extent, in which the traditional 1D ECG signal analysis method was abandoned, and instead, a 2D image method was employed. In the 2D ECG image, the *X*-axis represents time, and the *Y*-axis represents the amount of change in the ECG at each time point. The ResNet (He et al., 2016b; Somani et al., 2021) architecture was used to build the present feature model, and 2D ECG images were used as input training data. Related research has shown that ResNet is effective in capturing image features. Therefore, they have been widely used in computer vision and medical imaging (Rim et al., 2020). The network was relatively shallow before ResNet methodology was developed. When deeper networks were used, the performance was not good because it sometimes yielded poor results. The residual learning proposed by ResNet, which is adopted in the present study, makes it easier to train deep networks and opens the era of various ultradeep networks (Zaeemzadeh et al., 2021). In summary, the current model is a combination of ResNet and ANN, resulting in two types of components: a ResNet spatial component to solve 2D image problems, and an ANN model for personal information with the final feature of ECG waveform. This study shows that the proposed hybrid model is feasible for calculating BLC by integrating the advantages of two different types of neural networks.

### 4.3 The meaning of the proposed model

There is a need to understand the mechanics of the proposed model. Deep neural network models have the advantage of not requiring pre-information before modeling a system; therefore, their decision-making mechanism can be unclear to human cognition. It is often opaque and thus labelled a “black box.” Even so, many researchers have attempted to interpret deep neural network models to help people understand recognition mechanisms more intuitively. We not only built the BLC model but also attempted to preserve intuitive recognizable factors within the model. In the present model, system weighting can be seen as an intuitive understanding using the ANN model when the model is optimized.

Compared with our previous mathematical model (Huang et al., 2019), in which the HR and breathing rate (BR) are the main independent variables, the accuracy of the current model is greatly improved. The standard deviations of fitting error in the LM and H conditions are 0.12 and 0.19 mmol/L, respectively, in the present mathematical model, which was 0.52 and 1.82 mmol/L, respectively, in the previous model. The improvement in accuracy is possible because ECG encompasses not only HR and BR information (it is a known fact that the RR interval increases during expiration and decreases during inspiration) but also its change in the waveform in response to exercise, as described previously in the Introduction section.

### 4.4 Limitation

This study had several limitations. First, the sample size is relatively small. However, excellent fitting was attainable. Moreover, the fourfold cross-validation shows that the consistency of our data is good, and the mathematical model does not overfit. While one may question that additional data may be required to further refine the algorithm, the present study nonetheless demonstrates the feasibility of using the current model to estimate BLC during exercise. Secondly, it should be noted that the present study did not compare the experimental results with other AI methods. Our focus was not to determine which AI method is more suitable for these results, but rather to introduce a novel AI methodology for accurately predicting BLC using ECG data. Third, the algorithm obtained in this study may only be relevant to CWR cycling exercises. Studies should be conducted to test its validity during other exercise modalities (e.g., treadmill exercise). Fourth, no prospective validation procedures were performed. Nonetheless, if we perform a prospective validation, these data can be pulled into the learning model to generate a new algorithm. Fourfold cross-validation was already applied to our model to determine overfitting statistical learning.

## 5 Conclusion

This study is the first to demonstrate that BLC during exercise could be non-invasively acquired by calculating data from ECG. Through the use of 2D CNN-based combined with ANN-based methods, excellent accuracy was achieved. The implications of these experimental findings have potential applications in exercise training and testing.

## Data Availability

The raw data supporting the conclusion of this article will be made available by the authors, without undue reservation.
